# Relationship Transitions and Loneliness in Midlife and Older Adulthood

**DOI:** 10.1093/geroni/igaf122.371

**Published:** 2025-12-31

**Authors:** Iris Wahring, Urmimala Ghose, , NIlam Ram, Christiane Hoppmann, Denis Gerstorf

**Affiliations:** Humboldt Universität zu Berlin, Berlin, Berlin, Germany; Humboldt University in Berlin, Humboldt University in Berlin, Berlin, Germany; Stanford University, Stanford University, California, United States; UBC, Vancouver, British Columbia, Canada; Humboldt University Berlin, Humboldt University Berlin, Berlin, Germany

## Abstract

Prior research suggests that being married or living with a partner is linked to a lower likelihood of loneliness, with some studies indicating a stronger effect for men. Moreover, because social networks and their perception change across the lifespan, the impact of relationship status on loneliness may differ between middle-aged and older men and women. We investigated how three major relationship events—separation, moving in with a partner, and marriage—affect loneliness and whether these effects vary by gender and age. We also examined instances in which individuals both moved in and married within the same timeframe. Using data from 4,768 U.S. participants in the Health and Retirement Study (HRS), we applied propensity score matching to compare two-year changes in loneliness likelihood between individuals experiencing a relationship event and matched controls whose relationship status remained stable. Results showed that moving in with a partner was associated with a reduced likelihood of feeling lonely, particularly among older men who also married their partner within the same period. In contrast, marriage among already cohabiting individuals and separation were not linked to significant changes in loneliness. These findings highlight the protective role of relationship involvement against loneliness in midlife and older adulthood and underscore the importance of considering the co-occurrence of relationship events.

